# Caffeine in surface and wastewaters in Barbados, West Indies

**DOI:** 10.1186/s40064-015-0809-x

**Published:** 2015-02-03

**Authors:** Quincy A Edwards, Sergei M Kulikov, Leah D Garner-O’Neale

**Affiliations:** Department of Biological and Chemical Sciences, The University of the West Indies, Cave Hill Campus, PO BOX 64 Bridgetown, West Indies Barbados

**Keywords:** Barbados, Caffeine, Contamination, Surface water, Wastewater

## Abstract

Caffeine, a purine alkaloid drug, has been recognized as a contaminant of water bodies in various climatic regions, however, these environmental caffeine concentrations are the first to be reported in the tropical Caribbean. The major objective of this study was to develop an improved method to extract caffeine from surface and wastewaters in the warm Caribbean environment and measure caffeine concentrations in highly populated areas in Barbados. Caffeine was extracted from water via solid phase extraction (SPE); the acidified water samples were loaded onto C-18 cartridges and eluted with pure chloroform. The extracted caffeine was quantified using gas chromatography - mass spectroscopy - multiple reaction monitoring (GC-MS/MS-MRM). Method detection limits of 0.2 ng L^−1^ from 1 L water samples were achieved. Caffeine was detected in all environmental water samples investigated. The concentrations of caffeine in surface waters were detected in the range 0.1 - 6.9 μg L^−1^. The two wastewater treatment plants, primary and secondary treatment systems, significantly differed in their ability to eliminate caffeine in the raw sewage (38% and 99% caffeine removal efficiencies respectively). Thus, it may be essential to employ secondary treatment to effectively remove caffeine from wastewater systems in Barbados. Caffeine in water bodies are principally attributed to anthropogenic sources as caffeine-producing plants are not commonly grown on the island of Barbados. The study also shows the recalcitrance of caffeine to hydrolytic degradation.

## Introduction

Caffeine is one of the most widely consumed psychoactive substances in the world as it is consumed daily in coffee, tea, soft drinks and chocolate (Ferreira 2005). It is also an ingredient in condiments, tobacco and medications. Caffeine is popularly consumed as it is a stimulant of the central nervous system which has the effect of temporarily rejuvenating the body and restoring alertness (Ferreira 2005). The global average consumption of caffeine is estimated to be generally between 80 and 400 mg per person per day (Gokulakrishnan et al. 2005), but the average caffeine intake per person per day values for Switzerland, the United Kingdom and the United States of America are estimated at 300 mg, 440 mg and 210 mg respectively (Buerge et al. 2003; Standley et al. 2002). In humans caffeine is rapidly metabolized by the liver and the majority of the ingested caffeine is converted to one or more secondary metabolites. Thus, about 0.5% to 10% is excreted through urine and faeces (Seiler et al. 1999; Berthou et al. 1992; Knee et al. 2010; Rodriguez del Rey et al. 2012).

Although it is found in many types of plants, the presence of caffeine in environmental waters is largely attributed to discharges of domestic wastewater (Martín et al. 2012; Metcalfe et al. 2003; Seiler et al. 1999; Wu et al. 2010). On the island of Barbados caffeine plants are not commonly grown, moreover, few cocoa and coffee plants are found in botanical gardens. Some researchers have found that anthropogenic caffeine is transported to ponds and marine systems via streams and rivers draining to the coast. Other researchers have indicated that overflows of on-site wastewater treatment systems and storm water run-off are also contributors of caffeine to surface waters. (Buerge et al. 2006; Peeler et al. 2006; Rounds et al. 2009). However, a significant source of caffeine to domestic wastewater is likely to be the disposal of unconsumed coffee, tea or soft drinks down household drains and rinsing of coffee pots and cups. The disposal of even a few cups of coffee into household drainage systems could contribute to hundreds of milligrams of caffeine to surface waters as domestic wastewater from sinks may flow to streams, rivers or ponds that finally deposits into the marine environment (Seiler et al. 1999). Another anthropogenic source of caffeine to the environment that is commonly neglected is the direct disposal of leftover medications into household sinks, toilets, or in trash that end up at the landfill. Caffeine tracked the population density and elevated concentrations of caffeine were attributed to large populations (Peeler et al. 2006; Rodriguez del Rey et al. 2012).

Caffeine has several unique characteristics important for a good chemical marker of pollution. It is highly soluble in water (13 g L^−1^) having a very low octanol-water coefficient (log k_ow_ = −0.07), insignificant volatility and its half-life is about 10 years (Buerge et al. 2006; Lin et al. 2009; Froehner and Martins 2008; Chen et al. 2012). Kurissery et al. 2012 found that the concentration of caffeine did not show any significant correlation with hydrologic parameters such as surface water temperature, pH or dissolved oxygen indicating its stability and slow pace of degradation. It fits the profile for a good marker directly related to anthropogenic influences with no potential biogenic sources (Siegener and Chen 2002). However, researchers have proved that caffeine is readily biodegradable (Thomas and Foster 2005; Gómez et al. 2007).

Bacterial strains belonging to *Pseudomonas*, *Serratia*, and fungal strains of *Aspergillus*, *Penicillium*, *Phanerochaete*, *Rhizopus*, and *Stemphylium* are reported to degrade caffeine (Yu et al. 2009; Beltrán et al. 2006; Asano et al. 1993; Dash and Gummadi 2007; Yamaoka-Yano and Mazzafera 1999). Dash and Gummadi 2006 found that *Pseudomonas* is the best organism for caffeine degradation with *Pseudomonas* sp. NCIM 5235 showing complete degradation of 6.4 g L^−1^ of caffeine in 24 hours. Wastewater treatment plants (WWTPs) elimination of caffeine (81-100%) have been found to be more profound when secondary treatment (e.g. biological treatment) is employed (Buerge et al. 2006; Lin et al. 2009, Siegener and Chen 2002; Benotti and Brownawell 2007). Removal efficiency diminishes considerably in WWTPs using less advanced treatment processes (Rodriguez del Rey et al. 2012). For example, Boisvert et al. 2012 reported a removal efficiency of <10% using primary treatment in Canada.

Caffeine may also be a pollutant to marine ecosystems. A study by Pollack et al. 2009 suggested that exposure to caffeine may exacerbate the effects of other environmental parameters on coral such as changes in ocean temperatures and pH, making them more likely to undergo bleaching. Coral reef ecosystems are major aesthetic attractions in the Caribbean region and thus the sustainability of coral reefs in the region is important to our eco-tourism industry.

The study was instigated due to a lack of data on emerging contaminants in environmental waters in the Caribbean region. Based on our knowledge this investigation of caffeine or pharmaceuticals in surface and wastewater is the first to be conducted in the West Indies and may help to foster more research on emerging contaminants in the said region in the near future. Furthermore, in this part of the world there are no legislative guidelines associated with these contaminants in natural waters hence this research may help in the development of standards and regulatory testing of these compounds in our natural waters.

The primary aim of this work was the development of an integral methodology to determine the concentration of caffeine in surface and wastewater and evaluate the removal efficiencies of caffeine in two WWTPs in Barbados. These data are compared to results collected around the world. The secondary aims of the paper are to evaluate the sources of caffeine in surface water, whether natural or anthropogenic and assess the stability of caffeine in aqueous solutions.

## Materials and methods

### Site description

**Figure 1:**
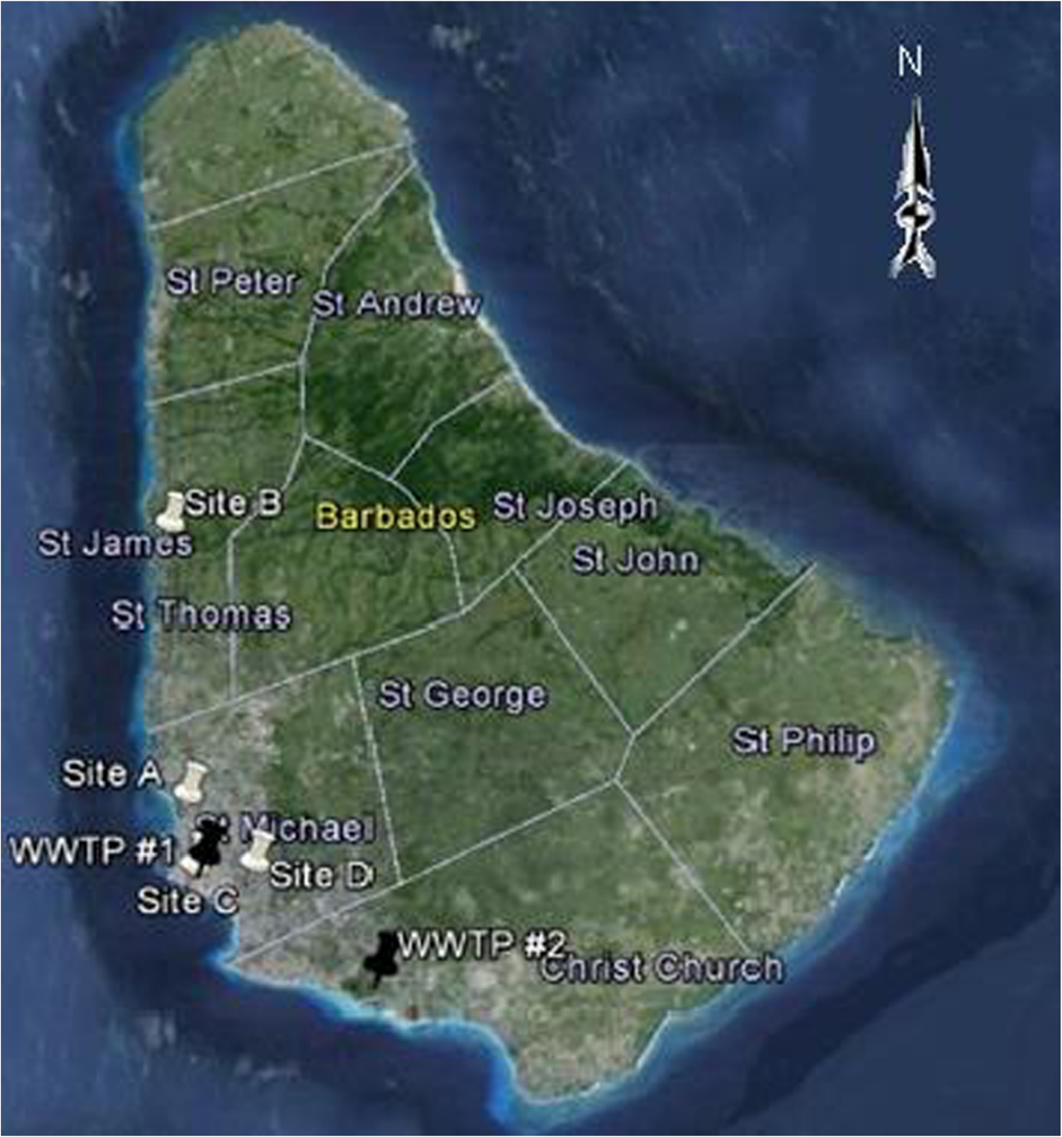
**Sampling sites in Barbados.** Black and white placemarks represent WWTPs and surface water sites respectively.

Both surface water and wastewater samples were collected during December 2012. The local mean temperature of December was 27°C. Barbados is 166 sq. miles (432 km^2^) and has a population of approximately 300,000 people. For this study four highly populated areas were selected for surface water sampling that represented both land drainage and various levels of anthropogenic influences including household, commercial businesses, and agriculture. These sites included Brandon’s Beach Catchment (Site A; N 13° 06′ 58.58″ W 59° 37′ 35.75″), Holetown Pond (Site B; N 13° 11′ 14.56″ W 59° 38′ 10.77″), Pelican Village Drainage (Site C; 13° 05′ 52.70″ W 59° 37′ 25.42″) and Queen’s Park Constitution River (Site D; N 13° 05′ 55.10″ W 59° 36′ 27.60″). Sites C and D are located in the capital city, Bridgetown in the parish of St. Michael, which inhabits about 120,000 people (7,300/sq.mile). Site A is also located in the parish of St. Michael but in a small urban area outside of Bridgetown inhabiting approximately 5,000 people (3,100/sq.mile). Site B is located in the town of the parish of St. James that has a population of about 30,000 people (2,400/sq.mile). The Holetown Pond, Site B, is a large body of water of approximately 80,000 gallons. All the surface water sites were fairly transparent and flowing with exception to site A which was stagnant, of green colour and a foul odour. All surface water runoffs are finally deposited at sea. Figure 1 shows a map of Barbados highlighting sampling sites in specific parishes.

Wastewater samples (both influent and effluent) were collected from the wastewater treatment plants (WWTPs) located in Bridgetown (9,500 - 11,300 m^3^/d, mainly commercial connections) and the South Coast (2,600 - 3,800 m^3^/d, mainly residential connections) of Barbados and analyzed for the target compound. The former plant, WWTP # 1 (N 13° 06′ 01.88″ W 59° 37′ 15.63″), is a secondary treatment plant serving community of Bridgetown (which is densely populated with a high concentration of local and commercial businesses) and other areas. The latter, WWTP # 2 (N 13° 04′ 20.64″ W 59° 34′ 22.97″), is a primary treatment plant also located in a highly populated area; however, the South Coast (Christ Church; 50,000 people, 2,500/sq.mil) is a tourist “hotspot” because of the abundance of hotels and night entertainment in that district. Domestic waste from hotels in the South Coast area is transported to WWTP # 2. The tourist season in Barbados extends from the month of December to April. Treated wastewater is also deposited at sea.

### Chemicals and materials

Natural caffeine 99.9% (Reagent Plus), chloroform (Chromaslov for HPLC), hydrochloric acid (HCl), methanol (Chromasolv) and sodium hydroxide (NaOH) were purchased from Sigma Aldrich (St. Louis, MO, USA). The internal standard 1,4-dichloro-benzene was obtained from BDH Laboratory (Poole, England). A stock solution of the standard was prepared separately in methanol at a concentration of 100 mg L^−1^. The stock solution was kept in the dark at 4°C. Caffeine surrogate internal standard (purity 99% ^13^C_3_-labelled, 100 μg mL^−1^) was later obtained from Cambridge Isotope Laboratories Inc. (Andover, MA, USA). Solid phase extraction cartridges used for sample preparation included Strata X (C-18 styrene-divinylbenzene polymer sorbent) cartridges (2 g) purchased from Phenomenex (Torrance, CA, USA). High Purity water (Sigma Aldrich, USA) was used for all experiments.

### Sampling and sample preparation

Grab water samples (1.2 L) were collected in 2012 from surface water and wastewater (both influent and effluent) sites during the dry season in highly populated areas in Barbados. Water samples were collected in amber glass bottles which were previously cleaned in the laboratory as followed: soap cleaned, rinsed five times with tap water, soaked in 10% hydrochloric acid for 1 hour, rinsed five times with distilled water and rinsed three times with methanol to remove any organic contamination. Each amber bottle was rinsed with sample water on the site immediately prior to collection. Samples were stored on ice (~4°C) and extracted within 24 hours.

Samples collected with sediments or algae were first centrifuged with a Beckman Centrifuge J2 MC (10,000 rpm at 25°C for 15 minutes) and then filtered via paper filtration under suction. Water samples (1 L) were acidified to pH 2 using HCl (1 Mol^−1^) and caffeine extracted via SPE Strata X cartridges. An improved SPE method was developed for the extraction of caffeine from natural water. The solid phase was conditioned with 15 mL of methanol followed by 30 mL of purified water adjusted to pH 2. Samples were passed through the cartridges at a flow rate of approximately 5 mL min^−1^. All sample bottles were rinsed three times with 10 mL of pH 2 purified water and the rinses were combined and passed through the SPE cartridge. The cartridge (sorbent) was then dried for 15 minutes under vacuum.

The caffeine analyte was eluted twice with two 50 mL portions of chloroform under vacuum at a flow rate of approximately 1 mL min^−1^ using a 10 fold manifold (Phenomenex, Torrance, CA, USA). After elution the cartridges were dried for 15 minutes. The solvent was immediately evaporated to dryness using a rotavapor rotary evaporator (Buchi Labortechnik AG, Switzerland; model RE-121) and reconstituted to a final volume of 1 mL in methanol and stored in amber vials. Reconstituted samples were stored in the refrigerator until analysis. Procedural blanks were run periodically to check for caffeine contamination during extraction. Recovery tests were made using purified water with caffeine concentrations of 0.5 and 1 μg L^−1^.

### Chromatographic analysis

A known concentration of internal standard, 1,4-dichlorobenzene, was added to the sample prior to GC-MS analysis. Aliquots of 1 μL, using an Agilent Technologies 7693 autosampler, of the final extracts were analyzed by gas chromatography coupled with mass spectroscopy (GC-MS) on an Agilent 7000A GC-MS triple quad model (USA). The extracts were injected in the splitless mode with the injector port temperature at 280°C. A 30 m × 0.25 mm i.d. DB-5MS (5% phenyl, 95% methylpolysiloxane) capillary column (Agilent, USA) with a 0.25 μm film thickness was used. Helium was used as the carrier gas at a constant flow of 1.2 mL min^−1^. The column temperature program for the GC oven was as follows: initial temperature 70°C, maintained for 2 minutes and then ramped at 20°C to 230°C, where it was held for 4 minutes. Total run time was 10 minutes.

The mass spectrometer with electron impact ionization at 70 electron volt (eV) and 25 eV in the first and second ionization chambers respectively was operated in multiple reaction monitoring (MRM) mode. The interface as well as the spectrometer transfer line temperature was maintained at 280°C. The peaks produced by caffeine and 1,4-dichlorobenzene were used to quantify natural caffeine. A multi-point calibration curve of peak area ratio (analyte to standard peak area) against analyte concentration was used to quantify the unknown concentration of the caffeine in field samples. The instrumental detection limit (LOD) and quantification limit (LOQ) were defined as the lowest analyte concentration that produced a peak with signal-to-noise ratio of 3 and 10 respectively (Li et al. 2010).

### Caffeine decomposition in aqueous solutions

The reaction kinetics was monitored by following the decomposition of caffeine in aqueous solutions over a variety of temperature and pH. Caffeine concentration was measured by GC-MS/MS-MRM as described above; however, standard addition of ^13^C_3_-labeled caffeine was added to the prepared sample prior to analysis. Reactions were carried out in a 250 mL glass reaction vessel with a water cool condenser at 100°C (boiling), unless stated otherwise. Caffeine solution (100 mL) of initial concentration of 25 mg L^−1^ was placed into the glass vessel; during the course of the reaction aliquots of the solution (2 mL) were taken and fast cooled with ice-water mixture. One millilitre of the cooled aliquot was diluted in methanol to give an initial 1 mg L^−1^ caffeine concentration at time zero. Experimental time varied from minutes to hours for acidic, neutral and basic solutions. Caffeine thermal decomposition reactions of temperatures 70-90°C were conducted in a Precision Thermo Scientific Oven (model 25 EM) that was pre-heated for 1 hour.

## Results and discussion

### Occurrence of caffeine in surface water

An improved SPE method was developed for the extraction of caffeine from water. Principally, chloroform was used for the elution of caffeine from the C-18 cartridge sorbent as opposed to a combination of MeOH and methylene chloride or methyl tert-butyl ether (MTBE) and MeOH used in earlier studies (Peeler et al. 2006; Verenitch and Mazumder 2008). Recovery tests showed that the developed SPE method was very effective (95-100%) in extracting caffeine from pure water, reported in Table 1. Solubility studies have found that caffeine is about twice as soluble in chloroform as opposed to methylene chloride and about one order of magnitude more soluble in chloroform than methanol at temperatures 27-32°C and thus chloroform should be an effective solvent to separate caffeine from solutions (Shalmashi and Golmohammad 2010). Dichlorobenzene was used as an internal standard in combination with external calibration due to the initial unavailability of ^13^C_3_ caffeine surrogate. Since this internal standard was only added prior to GC-MS analysis, this compound did not correct for extraction efficiencies, but was used as an indicator for instrument sensitivity and corrected for sample injection problems. Table 1 below shows the analytical parameters for the target compound.

**Table 1:** **Target compound and internal standards GC-MS and analytical parameters; chemical formulas, the chromatographic retention times, the mass spectrometric multiple reaction monitoring (MRM) ion transitions, external standard calibration linearity, the limits of detection (LOD) and quantification (LOQ) and percentage recovery of the analyte**

**Compound**	**Chemical formula**	**Retention time (min)**	**MRM transition**	**Linearity (R** ^**2**^ **)** ^**a**^	**LOD (ng L** ^**−1**^ **)**	**LOQ (ng L** ^**−1**^ **)**	**Recovery (%)**
Caffeine	C_8_H_10_N_4_O_2_	8.4	194 109	0.999(7)	0.2	1.0	95 - 100
Dichlorobenzene	C_6_H_4_Cl_2_	4.2	146 111	-	-	-	-
^13^C_3_ Caffeine	C_8_H_10_N_4_O_2_	8.4	197 111	-	-	-	-

^a^Number of points used for linear regression is in parenthesis.

^a^Linear range 0.1 - 50 μg L^−1^.

On the Caribbean island of Barbados caffeine-producing plants are rarely grown. Private conversations with local agronomists and botanists at the University of the West Indies revealed that only a few caffeine-producing plants are grown on the Island (cocoa and coffee plants) and the majority is housed in botanical gardens. Furthermore, caffeine plants were nowhere in sight during surface water sampling. Thus, caffeine levels in water bodies may principally be attributed to anthropogenic sources. Caffeine was found in all surface water sites investigated during the beginning (December) of the tourist season, and ranged from 0.1-6.9 μg L^−1^, shown in Table 2.

**Table 2:** **Mean (n = 3;**±**SD) concentrations (μg L** ^**−1**^ **) of caffeine in surface waters collected in Barbados**

**Locations**	**Caffeine (μg L** ^**−1**^ **)**
Site A: Brandon’s Beach Catchment	0.1±0.01
Site B: Holetown Pond	0.5±0.04
Site C: Pelican Village Drainage	0.4±0.04
Site D: Queen’s Park Constitution River	6.9 ±0.8

The average caffeine concentration at Site D was the highest (about 70 times greater than Site A) among the surface water sites investigated. This may be attributed to the fact that Site D is located in the “heart” of the capital city of Barbados (Bridgetown) which is the most densely populated area among the sites studied. Also, this river is in close proximity to densely populated residential areas and small business (e.g. convenient shops) that may be a non-point source of caffeine contamination as municipal wastes from sinks may be indirectly deposited into the river which drains into the marine environment. According to United States Environmental Protection Agency (US EPA) caffeine in domestic wastewater may be as a result of unconsumed coffee, tea or soft drinks disposed down household drains which may contribute to high concentrations of caffeine in surface water. Faulty septic systems and the disposal of medications into household sinks, toilets and trash are also relevant sources of caffeine to surface water. Furthermore, several researchers reported that caffeine concentrations in surface water positively correlated to the population density (Rodriguez del Rey et al. 2012; Martín et al. 2012; Froehner et al. 2010, Wu et al. 2010; Seiler et al. 1999).

Site C (a drainage stream) is also located in Bridgetown but at a different location that less transverse residential area. Proximal to site C are a few souvenir shops and small restaurants and bars that cater to cruise ship passengers as the ferry port is in walking distance. Thus, its location may contribute to the low (about 17 times less than site D) caffeine concentration found in this drainage stream or due to dilution, biodegradation or photodegradation. The caffeine levels at site B and C were similar, however, Site B is a surface water pond (approximately 80,000 gallons of water) located in the town of St. James which is the second most populated area examined. Thus, the caffeine concentration at Site B is highly diluted in comparison to site C. The pond is located in an upscale business community which is a tourist “hot spot” in Barbados. It is located in walking distance to the popular malls, cinema and night clubs. Therefore, the pond may potentially be exposed to caffeine from sodas or energy drinks sold at the cinema and night clubs or residential housing and commercial businesses run-offs via drainage pipes or during heavy rainfall. According to Rodriguez del Rey et al. 2012, storm water run-off maybe a source of caffeine to water bodies. Rounds et al. 2009, cautioned that caffeine might be present in streams as a result of people discarding beverages (e.g. coffee, sodas etc.) in the street. Thus, storm water could represent a significant source of caffeine to environmental waters.

Site A, the least populated area, was found to have the least caffeine (approximately 70 times less than Site D). This domestic wastewater stream catchment is located in a populated urban area outside of Bridgetown but within the St. Michael Parish. The drainage stream traverses a populated residential area and it is in close proximity to fast food restaurants and local pharmacies which are potential sources of caffeinated products. However, the water at this catchment was stagnant, notably had an unpleasant smell and with an abundance of algae, characterized by the green colour of the water sample. In contrast, the water samples collected at the other surface water sites were fairly transparent and flowing. The low caffeine concentration in the catchment is likely due to biological activities and solar degradation of caffeine. Biological degradation within a water system is dependent on many factors such as microbial activity, temperature, trophic conditions and water depth (Buerge et al. 2003). The wastewater in the catchment was shallow and since the typical atmospheric temperature in Barbados ranged from 26–29°C, biological decomposition along with solar degradation are likely to destroy the caffeine present in the Brandon’s Beach Catchment. Several bacteria (e.g. *Serratia* and *Pseudomonas*), fungi (e.g. *Aspergillus*, *Penicillium*, *Stemphylium*) and algae (e.g. *Symbiodinium* clades: *Aiptasia* and *Pseudoterogorgia*) have been found to utilize caffeine as a carbon source (Pollack et al. 2009; Yu et al. 2009; Beltrán et al. 2006; Asano et al. 1993; Dash and Gummadi 2007; Yamaoka-Yano and Mazzafera 1999). All surface water sites investigated drains into the ocean. Thus, caffeine in the marine environment may also increase the population of microbes in seawater.

**Figure 2:**
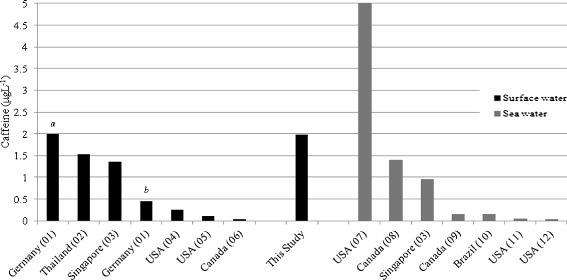
**Selected average concentrations of caffeine in surface waters collected worldwide.** Letters in italics represent locations proximal to wastewater plants: *a* downstream, *b* upstream. Numbers in parentheses are the following references: *01* Bahlmann et al. 2012, *02* Tewari et al. 2013, *03* Wu et al. 2010, *04* Siegener and Chen 2002, *05* Knee et al. 2010, *06* Yargeau et al. 2007, *07* Benotti and Brownawell 2007, *08* Comeau et al. 2008, *09* Verenitch and Mazumder 2008, *10* Ferreira 2005, *11* Rodriguez del Rey et al. 2012, *12* Singh et al. 2010.

**Figure 3:**
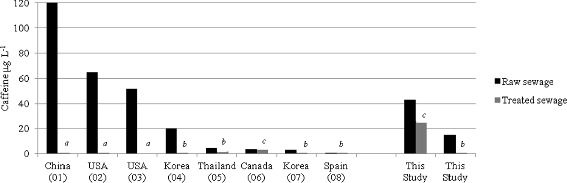
**Selected average concentrations of caffeine in wastewater samples collected worldwide.** Letters in italics are the following types of wastewater treatment effluents: *a* tertiary, *b* secondary, *c* primary. Numbers in parentheses are the following references: *01* Yang et al. 2011, *02* Trenholm et al. 2006, *03* Karnjanapiboonwong et al. 2011, *04* Sim et al. 2011, *05* Tewari et al. 2013, *06* Boisvert et al. 2012, *07* Behera et al. 2011, *08* Martín et al. 2012.

It was interesting to observe that the average caffeine concentration (6.9 μg L^−1^) at Site D was notably the highest concentration found in surface water when compared to literature findings. In most cases it was two to four times greater than surface water sites worldwide (Figure 2). In addition, the caffeine concentration at Site D exceeded that of raw sewage found in Canada, Korea, Spain and Thailand (Figure 3). This could be attributed to the fact that the volume of water at site D is relatively low (less diluted) as compared to the international sites mentioned. Importantly, the average caffeine concentration in surface water in Barbados based on the four sites investigated is about 2 μg L^−1^ as shown in Figure 2. The average caffeine concentration is similar to that of Germany. The preliminary results of this study may suggest that caffeine in surface water in Barbados relates to the population density as greater levels of caffeine in surface water are found in the more populated areas that may originate from anthropogenic sources.

To date no conclusive study has been done on caffeine as a contaminant and based on our knowledge there are no established guidelines for caffeine concentrations in environmental waters. However, the United States Food and Drug Association (U.S. FDA) requires environmental risk assessments of pharmaceutical compounds if predicted introductory concentrations are expected to be greater than 1 μg L^−1^ (United States Federal Drug Administration 1998). In contrast, the European Union (EU) mandates environmental risk assessment of all pharmaceutical substances with a predicted environmental concentration (PEC) of 0.01 μg L^−1^ (O’Brien and Dietrich 2004). The caffeine concentration found in Queen’s Park Constitution River (Site D) is about seven (7) times and two orders magnitude greater than the U.S. FDA and EU PEC limits respectively for pharmaceutical compounds in environmental water and should be a “cause for concern” as the river drains to the marine environment. Literature findings have found caffeine in coastal waters ranging between 0.001-5 μg L^−1^. Furthermore, caffeine in seawater has only recently being recognized of having potential effects on marine ecosystems. In one of the only studies to investigate the effect of caffeine on coral reefs, Pollack et al. 2009 alerted that caffeine may increase the effects of other environmental factors on corals and making them more susceptible to bleaching. Barbados, the Caribbean region, is known for its vibrant marine eco-tourism (e.g. scuba diving, submarine tours etc.) and lucrative fishing industry. Moreover, many individuals are employed in this fishing industry and thus healthy coral reefs are essential in maintaining the livelihood of these individuals in the region. It is of utmost importance for the region to monitor caffeine and other contaminants in surface water and the marine environment.

### Occurrence of caffeine in WWTPs

As expected the caffeine concentrations in raw sewage was much greater than that found in surface waters in Barbados. The concentration of caffeine varied at both wastewater treatment facilities with the South Coast plant (WWTP 2) having higher concentrations of caffeine (about three times more caffeine) than the Bridgetown plant (WWTP 1) as shown in Table 3.

**Table 3:** **Mean (n = 3;**±**SD) concentrations (μg L** ^**−1**^ **) of caffeine in wastewater collected from WWTPs that serve Barbadian communities**

**Wastewater treatment plants**	**Caffeine (μg L** ^**−1**^ **)**	**Efficiency (%)**
1: Bridgetown		
Influent	15±1.2	99
Effluent	0.1±0.01	
2: South Coast		
Influent	43 ±2.9	38
Effluent	26 ±1.3	

The WWTP 1 was found to be very effective in eliminating caffeine (99% efficiency). On the other hand, the WWTP 2 was not as effective (38% efficiency). The difference in the caffeine removal efficiencies at each wastewater plant could largely be due to the fact that the Bridgetown wastewater plant is a secondary treatment plant (removing all suspended and dissolved solids by combining them with activated sludge) whereas the South Coast wastewater plant is a primary treatment system (aeration and filtration). Thus, the Bridgetown plant is essentially the only system of the two that treats the wastewater, hence the significant difference in effluent quality. In addition, the South Coast sewage treatment plant (STP) serves commercial enterprises (hotels etc.) with approximately four thousand (4,000) connections but the majority of waste received is residential. The Bridgetown plant services about one eighth of the capital and its immediate environment with approximately one thousand five hundred (1,500) connections majority being commercial. The average daily flow rate for the Bridgetown and South Coast sewage systems are 2.75 millions of gallons per day (mgd) and 0.85 mgd respectively. The higher concentrations of caffeine found in the influent at the South Coast STP than the Bridgetown STP may be as a result of the former receiving a greater volume of waste daily and/or the population (including tourists) on the South Coast of Barbados are consuming higher volumes of caffeinated products (e.g. coffee). It is important to note, the South Coast STP is located in the Christ Church parish that has numerous hotels and night clubs and as a result the volume of raw sewage being carried to this treatment plant might be much greater than the raw sewage being carried to the Bridgetown plant daily. Furthermore, sampling was conducted at the beginning (December) of the tourist season. Generally during the month of December there is a vast increase in numbers of stay over visitors in Barbados.

The caffeine profile diagram (Figure 3) above shows that secondary and tertiary wastewater treatments are very effective in eliminating caffeine. According to Figure 3 below, advance (tertiary) treatment plants consisting of biological treatment (e.g. activated sludge and biological reactor processes) along with chlorination or ozonation produced caffeine-removing efficiencies of 98-100% (Yang et al. 2011; Trenholm et al. 2006; Karnjanapiboonwong et al. 2011). Secondary treatment plants that employed primary treatment along with biological treatment produced caffeine-removing efficiencies of 70-96% (Sim et al. 2011; Tewari et al. 2013, Behera et al. 2011; Martín et al. 2012). In Canada, primary sedimentary tanks were ineffective in eliminating caffeine resulting in < 10% removal efficiency (Boisvert et al. 2012).

The caffeine removal efficiency results in this study were consistent with that of the literature as the secondary treatment plant (99%) was very effective in eliminating caffeine. However, the primary treatment plant (38%) was less effective in destroying caffeine. There is a paucity of data related to caffeine removal in primary treatment systems. Thus, in relation to the research conducted in Canada by Boisvert et al. 2012 the primary treatment plant in Barbados was found to be more effective in eliminating caffeine. Aeration of the raw sewage at the primary treatment plant in Barbados may have allowed for some aerobic microbial decomposition of caffeine or due to some adsorption of caffeine to sludge particles. Other significant factors such as residence time, flow rate and sludge age must also be considered. Several researchers maintained that wastewater treatment plants elimination of caffeine (81-100%) have been found to be most effective when secondary treatment (e.g. biological treatment) is employed (Buerge et al. 2006; Lin et al. 2009; Siegener and Chen 2002; Benotti and Brownawell 2007).

In Barbados the treated wastewater is deposited far out at sea. The U.S. FDA recommended a 10-fold dilution factor to predict the concentrations of drugs in surface waters from estimated concentrations in STP effluents (United States Federal Drug Administration 1998). Moreover, researchers have found that this dilution factor is representative of effluent discharges into natural water (Metcalfe et al. 2003; Dorn 1996). Future research on caffeine in marine environment in Barbados will determine if the FDA 10-fold dilution factor is representative of caffeine concentrations in STP effluents deposited at sea.

### Caffeine decomposition in aqueous solutions

#### Acidic and neutral media

Concentration of caffeine in pure water and 0.01 mol L^−1^ HCl aqueous solutions did not show any evidence of decomposition after boiling at 100°C under reflux for 8 hours. The stability of caffeine at acidic and neutral pH may be due to its aromaticity. Caffeine is a two member ring structure consisting of amides, amines and alkenes functional groups. The five-membered ring in the caffeine molecule is aromatic as it is cyclic and planar. Furthermore, the N-CH_3_ nitrogens have a lone pair located in the p-orbital that can participate in delocalization and resonance which increase the stability of the molecule. According to Boisvert et al. 2012, caffeine is in its protonated form at pH conditions below its pka of 10.4. At alkaline conditions greater than pH 10.4, the molecule is found in its neutral form. Researchers have maintained that the protonated form is more water soluble and less volatile than the neutral, unprotonated form (Boisvert et al. 2012; Bahrami et al. 2013). Therefore, it may be impossible to study the stability of caffeine to hydrolysis at conditions close to the natural environment in Barbados (neutral solutions and temperatures 26-30°C). However, caffeine in 0.01 mol L^−1^ NaOH slowly degraded at 100°C. This proved that base catalyzed reaction kinetics could be studied at elevated NaOH concentrations. Furthermore, the results can be extrapolated to fit normal temperatures and variable pH.

#### Basic solutions

Experimental data shows that the rate of caffeine disappearance is first order with respect to concentration of both caffeine and OH ^−^. The second order rate constant at 100°C was found to be 0.71 ± 0.10 mol L^−1^ min^−1^. This shows that we can estimate the rate constant and half-life of caffeine destruction at moderate pH and temperature with reasonable accuracy.

1 $$ \frac{\hbox{-} \mathrm{d}\left[\mathrm{Caffeine}\right]\ }{\mathrm{dt}} = {\mathrm{k}}_2\left[\mathrm{Caffeine}\right]\ \left[\mathrm{O}{\mathrm{H}}^{\hbox{-}}\right] $$

2 $$ {\mathrm{k}}_{1\left( \exp \right)} = \kern0.37em \left[\mathrm{O}{\mathrm{H}}^{\hbox{-}}\right] \times {\mathrm{k}}_2 $$

At constant NaOH concentration, pseudo-first order rate constant k_exp_ = k[OH^−^] can be calculated from the integrated rate equation:

3 $$ \ln\ \left[\mathrm{Caffeine}\right] = \hbox{-} {\mathrm{k}}_{\exp}\;\mathrm{t} + \mathrm{constant} $$

**Figure 4:**
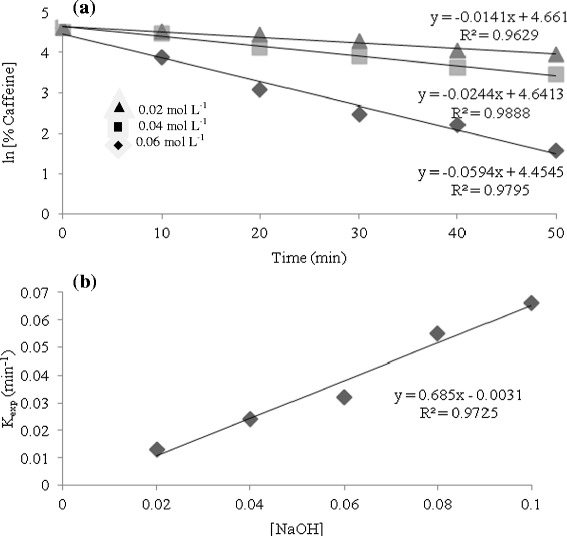
**Effects of different NaOH concentrations on (a) caffeine degradation at 100°C and (b) the overall rate of degradation.**

In correspondence with equation 3, Figure 4 illustrates the kinetics of caffeine disappearance in the range of basic concentrations investigated. The experimental rate constants and half-lives at 100°C are presented in Table 4.

**Table 4:** **Rate constants and half-lives for the destruction of caffeine in solutions of varying [OH] at 100°C**

**[NaOH] mol L** ^**−1**^	**k**_**exp**_ **(min** ^**−1**^ **)**	**Half-life (min)**
0.02	0.014	49.5
0.04	0.024	28.9
0.06	0.052	13.3
0.08	0.059	11.7
0.1	0.066	10.5

**Figure 5:**
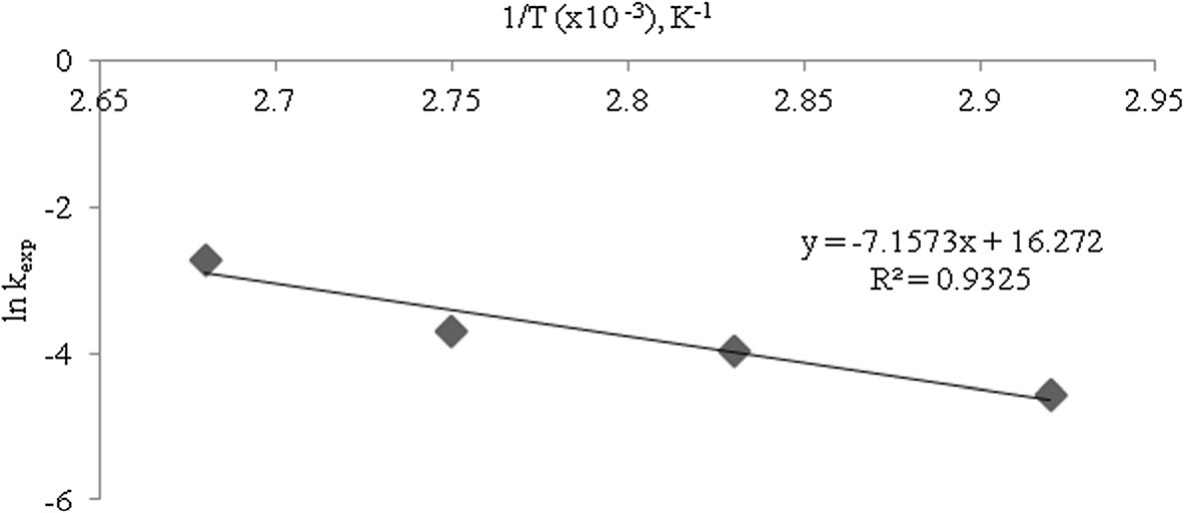
**Arrhenius plot for decomposition of caffeine; Ea = 59.5 kJ mol** ^**−1**^ **and A = 1.2×10** ^**7**^ **min** ^**−1**^ **.**

The data shows that caffeine should be destroyed (whether decomposed, transformed or mineralized) within minutes to hours when subjected to strong alkaline conditions and elevated temperatures. However, even more interesting is its estimated rate of degradation at conditions close to the natural environment in the Caribbean region. Using the Arrhenius plot (Figure 5), Arrhenius equation was used to determine the rate of caffeine degradation at 30°C. The results are reported in Table 5.

**Table 5:** **Calculated rate constants and half-lifes for the disappearance of caffeine in water at 30°C, extrapolated from experimental data**

**pH**	**k (min** ^**−1**^ **)**	**Half-life (Years)**
6	6.6×10^−11^	2.0×10^4^
7	6.6×10^−10^	2.0×10^3^
8	6.6×10^−9^	2.0×10^2^
9	6.6×10^−8^	2.0×10^1^

Calculations show that caffeine should be practically stable indefinitely in aqueous solutions at moderate temperature and pH. Environmental water in Barbados (Caribbean region) is generally of pH 6–8. Notably, this estimation does not take into account biological degradation, dilution, sorption or ultraviolent radiation (UV). These other means of caffeine destruction or chemical transformation in the environment are important, however, researchers emphasized that partitioning (sedimentation and volatilization) and photodegradation have negligible effects on caffeine in surface waters. Biodegradation is probably the principal means of caffeine removal from surface waters (Buerge et al. 2003; Buerge et al. 2006).

The stability of caffeine at moderate temperature and pH is in agreement with Buerge et al. 2006 who concluded that caffeine degrades slowly (half-life of about 10 years). However, we have found its half-life between 20–20,000 years depending on the pH of water.

**Figure 6:**
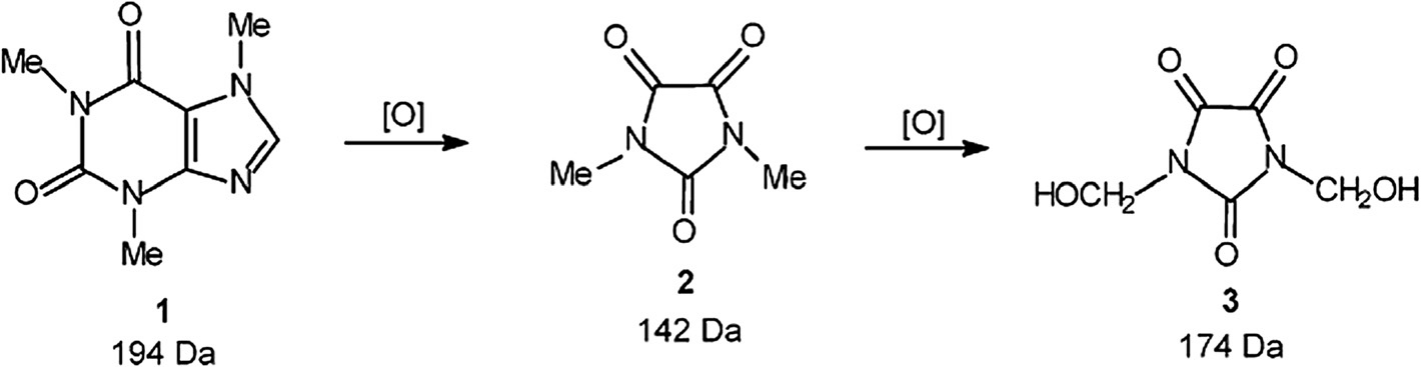
**Caffeine and its oxidative by-products (Dalmázio et al.** 2005 **).**

Dalmázio et al. 2005, emphasized that caffeine (194 Da) degradation is likely to generate persistent intermediates that may not be as efficiently oxidized as caffeine. In their work they highlighted two major degradation products (*N*- dimethylparabanic acid, 142 Da, and di(*N*-hydroxymethyl) parabanic acid, 174 Da) generated by advanced oxidative conditions. Furthermore, the researchers showed the importance of the hydroxyl radical in caffeine destruction or transformation leading to slow mineralization. Caffeine is oxidized by hydroxyl radicals in water as shown in Figure 6.

Other studies have identified other caffeine intermediates via demethylation, hydrolysis and hydroxylation. These included 1,3,7-trimethyluric acid, theophyline, 1,3-dimethyluric acid and 6-amino-5-(*N*-formylmethyl-amino)-1,3-dimethyl-uracil (Telo and Vieira 1997; Stadler et al. 1996).

The results of this study corroborate the recalcitrance of caffeine to hydrolytic degradation in water of moderate temperature and pH. Thus, caffeine disappearance (whether transformation or mineralization) in the environmental water may be due to other processes such as biodegradation, dilution, sorption and solar destruction.

## Conclusions

The developed SPE method was successful in extracting caffeine from environmental water. GC-MS/MS-MRM was effective as a quantification method for caffeine in water samples at ng L^−1^ concentrations. The study has identified caffeine in all surface water sites investigated and has highlighted one specific site in the capital city having caffeine concentration above those found in EU and U.S. surface waters. Caffeine levels in surface water are primarily attributed to anthropogenic sources (caffeinated products) as caffeine-producing plants are rare in Barbados. Caffeine was found in higher concentrations at the South Coast WTTP than the Bridgetown WWTP. However, the latter (secondary treatment plant) was more effective in eliminating caffeine than the former (primary treatment plant). The caffeine concentrations in raw sewage and treated sewage are similar to samples collected elsewhere. All surface water sites and treated wastewater are disposed at sea.

Little is known about the effects of caffeine in the marine waters; however, some studies have cautioned that caffeine in the seawater may exacerbate the effects of other environmental factors that aid in coral bleaching. This study has found caffeine to be practically stable indefinitely in aqueous solutions thus its destruction in the environment is due to other processes. Currently, the Caribbean region does not have established standards or health advisories for caffeine and pharmaceuticals in natural waters. Thus, more research on caffeine as a potential chemical indicator of domestic wastewater may become an important issue because of its clear anthropogenic origin and ubiquitous detection in surface and wastewaters.
